# A fast, simple and robust protocol for growing crystals in the lipidic cubic phase

**DOI:** 10.1107/S0021889812037880

**Published:** 2012-10-10

**Authors:** Margaret Aherne, Joseph A. Lyons, Martin Caffrey

**Affiliations:** a Membrane Structural and Functional Biology Group, Schools of Medicine and Biochemistry and Immunology, Trinity College Dublin, Ireland

**Keywords:** crystallization, *in meso* crystallogenesis, lipids, membrane proteins, mesophase, receptors

## Abstract

A simple and inexpensive protocol for producing crystals in the sticky and viscous mesophase used for membrane protein crystallization by the *in meso* method is described. It provides crystals that appear within 15–30 min of setup at 293 K. The protocol gives the experimenter a convenient way of gaining familiarity and a level of comfort with the lipidic cubic mesophase, which can be daunting as a material when first encountered. Having used the protocol to produce crystals of the test protein, lysozyme, the experimenter can proceed with confidence to apply the method to more valuable membrane (and soluble) protein targets. The glass sandwich plates prepared using this robust protocol can further be used to practice harvesting and snap-cooling of *in meso*-grown crystals, to explore diffraction data collection with mesophase-embedded crystals, and for an assortment of quality control and calibration applications when used in combination with a crystallization robot.

## Abstract 2

A simple robust manual protocol for producing crystals in the lipidic cubic phase in less than an hour is described. It is designed to provide newcomers to the *in meso* method for crystallizing membrane proteins with experience of preparing, handling and growing crystals in the sticky and viscous lipidic mesophase.

## 1. Introduction

**Figure 1:**
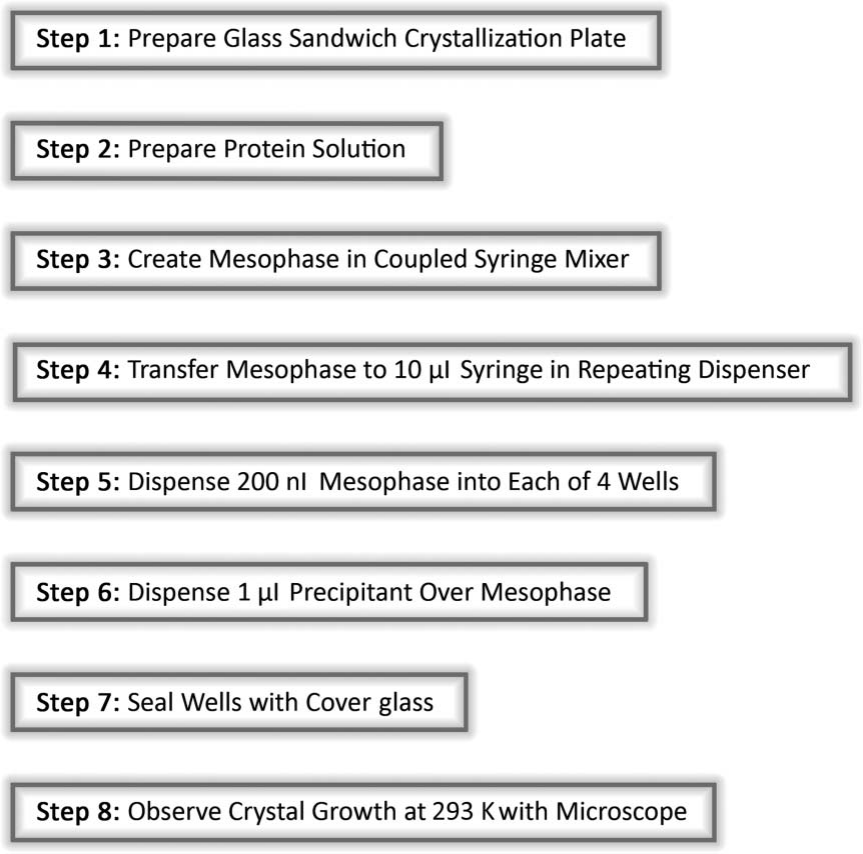
Steps involved in manually setting up *in meso* crystallization trials with lysozyme following a protocol that produces crystals within 30 min at 293 K. An open-access video of the protocol can be viewed online (Caffrey & Porter, 2010 ▶).

Crystallizing membrane proteins in lipidic mesophases is an established and proven method (Caffrey *et al.*, 2012 ▶). It is responsible for one-tenth of the membrane protein structures in the Protein Data Bank (http://www.rcsb.org/pdb/). Among these are some high-profile structures; the β_2_-adrenoreceptor–Gs protein complex is an example (Rasmussen *et al.*, 2011 ▶). Despite its many successes (Raman *et al.*, 2006 ▶; http://www.mpdb.tcd.ie), the method is being used in surprisingly few laboratories worldwide. The poor uptake in the community is probably attributable to the stickiness and viscosity of the mesophase in which crystallization occurs. In this laboratory note we provide a protocol that allows for the simple and inexpensive setting up of crystallization trials by the so-called *in meso* method, which produces crystals readily visible with a light microscope within an hour. Lysozyme was chosen as the test protein because it is available commercially at low cost, it is water soluble, stable and colourless, and it crystallizes *in meso* with great ease and alacrity (Landau *et al.*, 1997 ▶; Tanaka *et al.*, 2004 ▶). It is not a membrane protein. However, the focus of this note is not the target protein itself. Rather, the aim is to provide the experimenter with the experience of preparing and handling the sticky viscous mesophase in a way that can be replicated with what is likely to be a considerably more valuable membrane protein. Thus, the learning can be done and mistakes made with material that mimics the ‘real thing’ but that comes at a fraction of the cost. This note takes the user through the process of preparing special glass sandwich plates, producing the protein-laden mesophase, manually dispensing the mesophase into wells on the glass plate and observing colourless crystals as they grow in matters of minutes (Fig. 1 ▶). The protocol is simple, proven and robust.

As soon as the user has mastered the method and has gained familiarity with the challenging rheology of the cubic mesophase, trials with a *bona fide* membrane protein target can commence in the knowledge and with the confidence that the method, in that experimenter’s hands, does work. That confidence will carry the user through the many rounds of screening trails and optimizations often required in the quest for diffraction-quality crystals and a high-resolution structure.

## 2. Materials and methods

### 2.1. Materials

Lyophilized chicken egg-white lysozyme (CEWL; product No. L6876, lot 114K0626), sodium acetate (product No. S2889, lot 066K0043), hydrochloric acid (product No. 320331, lot S77015-109) and PEG 400 (product No. Fluka 81172, lot 1421464) were obtained from Sigma–Aldrich (Dublin, Ireland). Sodium chloride (product No. BP358, lot 097127) was obtained from Fisher Scientific (Dublin, Ireland). Monoolein (product No. M239, lot M239-F15-U) was purchased from Nu Check Prep Inc. (Elysian, MN, USA). Water, with a resistivity of >18 MΩ cm, was purified using a Milli-Q Water System (Millipore, Bedfors, MA, USA) consisting of an Elix 5 UV compartment (lot F4HN34349) with a Prograd2 cartridge (lot F1EA63837) to pre-purify the water and a Synergy compartment (lot F4EN79695B) with a Simpak1 cartridge (lot F1DA56566) to produce highly purified water, which was then treated by sterile filtration through a 0.22 µm MilliPAK40 filter (lot F5PN18060). Hamilton gastight syringes [model No. 1710N 100 µl syringe with cemented needle, 1710RN 100 µl syringe with removable needle (RN) connection, and 1701RN 10 µl syringe], Hamilton removable needles (22 gauge/13.3 mm/Blunt Style 2, custom made, product No. 7804-01) and a Hamilton PB-600-1 repeating dispenser device (product No. 83700) were obtained from Fisher Scientific (Dublin, Ireland). Syringe filters (0.45 µm, Filtropur S 83.1826 and 0.20 µm, Filtropur S 83.1826.001) were obtained from Sarstedt (Wexford, Ireland).

### 2.2. Crystallization plates

The plates for setting up crystallization trials were prepared with standard (26 × 76 mm) glass microscope slides as base plates (Caffrey & Cherezov, 2009 ▶). If untreated microscope slides (VWR, product No. 631-1550) are used they can be silanized simply by following the instructions on any commercially available silanizing solution (for example, Rain-X, Shell Car Care International, stock No. 80199200) commonly used to coat car windscreens. Double-sided spacer tape, 140 µm thick with 6 mm-diameter perforations (Saunders, a division of R. S. Hughes Co. Inc., 3M Tape 9500PC), was positioned on the glass slide to create the wells, as described by Caffrey & Cherezov (2009 ▶).

### 2.3. Precipitant

The precipitant solution consisted of 1 *M* sodium chloride, 0.1 *M* sodium acetate buffer pH 4.5 and 30%(*v*/*v*) PEG 400. Precipitant was prepared by combining appropriate volumes of the following stock solutions: 4 *M* sodium chloride, 1 *M* sodium acetate adjusted to pH 4.5 with 37%(*v*/*v*) hydrochloric acid and 50%(*v*/*v*) PEG 400. The sodium chloride and sodium acetate stocks were clarified using 0.45 µm syringe filters. To facilitate preparation of the polymer stock solution, solid PEG 400 stored at 277 K was placed for 20 min in a water bath at 328 K.

### 2.4. Protein

The protein solution was prepared with CEWL at a concentration of 50 mg of protein per millilitre in milli-Q water immediately before use in setting up trials. Having lysozyme powder pre-weighed in 25–50 mg quantities in a 1 ml Eppendorf tube facilitates solution preparation, which then simply requires the addition of 0.5–1 ml of milli-Q water followed by mixing. If necessary, the protein solution can be stored on ice until needed for mesophase preparation.

### 2.5. Mesophase

The mesophase was prepared by combining the hosting lipid, monoolein, with the lysozyme solution in coupled 0.1 ml gastight RN-type microsyringes in a volume ratio of 3/2, as described previously (Cheng *et al.*, 1998 ▶; Caffrey & Cherezov, 2009 ▶). The monoolein was first melted at ∼318 K to facilitate loading it, as an oil, into the microsyringe. This operation benefits from (i) working at an ambient temperature above 293 K, (ii) warming the syringe slightly (to ∼298 K) ahead of filling with molten lipid and (iii) using a lysozyme solution that has been equilibrated to ≥295 K. If the ambient temperature is below 293 K then the lipid can turn waxy, which makes handling more difficult. An open-access video is available online, which shows how to load the coupled syringe mixing device and to effect mixing of lipid and protein solution to produce the homogenous mesophase (Caffrey & Porter, 2010 ▶). The mesophase achieves a characteristic smooth viscous texture and is optically clear and isotropic (nonbirefringent) when formed properly. The protein-laden mesophase once formed is transferred to a 10 µl microsyringe mounted in a repeating dispenser device, as described previously (Caffrey & Cherezov, 2009 ▶). The dispensing syringe is fitted with a 13.3 mm-long 22 gauge needle for convenient delivery of the mesophase into crystallization wells. With this configuration, a single activation of the button on the repeating dispenser delivers a bolus of mesophase that measures ∼200 nl. It is, of course, possible to use a 1 or 5 µl dispensing syringe, in which case the dispensed mesophase volume will be 20 or 100 nl, respectively. A modified repeating dispenser is available for use with even smaller volumes (Cherezov & Caffrey, 2005 ▶).

### 2.6. *In meso* crystallization

Crystallization trials were set up by hand in glass sandwich plates using 200 nl of the protein-laden mesophase and 1 µl of precipitant solution per well, as previously described (Caffrey & Cherezov, 2009 ▶; Caffrey & Porter, 2010 ▶). This involves loading sequentially four adjacent wells with mesophase and immediately covering each bolus with 1 µl of precipitant solution using a 2 µl micropipette (P2, Gilson). As quickly as possible, a standard 22 × 22 mm glass cover-slide (product No. 831-0123 VWR) is placed over the four wells and is tamped firmly and uniformly in place on the double-sided tape with a small screwdriver or spatula to create a hermetic seal. The process just described is repeated until all wells on the plate are filled and sealed.

Plates were stored at 293 K and crystallization was monitored by using a light microscope (Nikon Eclipse E400 POL, Nikon Instruments Europe BV), taking care that the plate did not warm up on the stage of the microscope. If an incubator/imager, set to 293 K, is available it can be used conveniently to follow crystallization. Crystals begin to form within 15 min and are clearly visible and well developed within an hour. Viewing the plate between crossed polarizers on the microscope reveals crystals as bright objects on a dark background (note that the cubic mesophase is optically isotropic and nonbirefringent). Following this protocol, crystals reach a maximum size of 25–30 µm after 4–5 h and are stable at these dimensions for 2–3 d. Upon storage beyond that time the crystals slowly dissolve and may disappear. To track the course of crystal growth in this study digital images were recorded using an imager (RI1500, Formulatrix, Waltham, MA, USA).

## 3. Results and discussion

**Figure 2:**
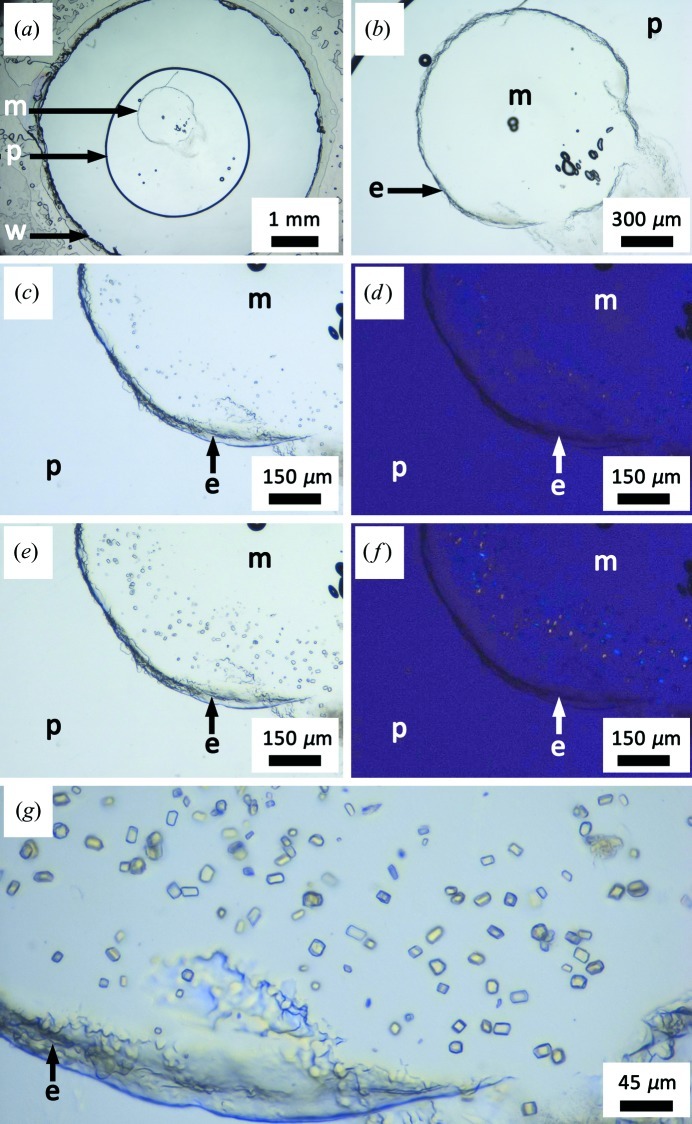
Crystallization of lysozyme at 293 K in the lipidic cubic phase prepared with monoolein as the hosting lipid. (*a*) A view of a suitably positioned 200 nl mesophase (m) bolus surrounded by 1 µl of precipitant (p) solution in a 6 mm-diameter well (w). (*b*) A close-up view in normal light of the mesophase immediately upon setup. The edge (e) of the mesophase bolus is marked with an arrow. (*c*) An expanded view in normal light of the mesophase 30 min after setup. Small crystals are obvious as dark flecks around the perimeter of the bolus. (*d*) As in (*c*), viewed between crossed polarizers. (*e*) An expanded view of the mesophase in normal light 60 min after setup. Crystals are clearly visible as dark flecks around the perimeter of the bolus. (*f*) As in (*e*), viewed between crossed polarizers. Crystals are apparent as bright flecks on a dark background. (*g*) An expanded view in normal light of crystals growing in the lipidic mesophase 85 min after setup.

The protocol introduced above will produce clearly visible lysozyme crystals in the lipidic mesophase within 60 min (Fig. 2 ▶). Microcrystals are apparent in the first 15 min of setup and usually begin to form along the perimeter of the mesophase bolus (Figs. 2 ▶ *a*–2 ▶ *f*). With time, as the precipitant diffuses into the core of the bolus, the entire volume of mesophase shows evidence of crystal growth. Further growth can happen beyond the first hour but usually stops after five hours. Crystals remain in place and of fixed size for 2–3 d but, over time at 293 K, they disappear as the protein diffuses out of the porous mesophase into the bathing precipitant solution.

For the experimenter wishing to gain experience handling the mesophase, with a view to using it for more valuable (membrane) protein crystallization trials, it is important to demonstrate that lysozyme crystals of the type shown in Fig. 2 ▶ can be generated reproducibly. Further, the mesophase bolus and precipitant solutions should be centred in the well without the precipitant touching the well wall, as in Fig. 2 ▶(*a*). If the bolus has been properly dispensed and the cover slip suitably positioned to seal the well, the bolus should take on the appearance of a circular disc, 140 µm thick (the spacer tape thickness), with the edge slightly roughened, as in Figs. 2 ▶(*b*)–2 ▶(*g*). The rough edging is characteristic of the viscous cubic mesophase, which does not flow readily. Checking that the crystals light up on a dark background when the plate is viewed between crossed polarizers, as in Figs. 2 ▶(*d*) and 2 ▶(*f*), provides convincing evidence that the protocol has worked and that crystals are indeed growing in the optically isotropic cubic mesophase.

The protocol in this laboratory note is being used by the senior author in demonstrations of the *in meso* crystallization method conducted at schools and workshops worldwide. As described, the protocol is simple, robust (in that it travels well) and produces crystals reliably within 30–60 min of setup. As part of these demonstrations it is common to have the students set up their own plates. In such situations also, the protocol works reliably and crystals grow. Accordingly, it should work for others conducting similar courses and workshops devoted to the practice of macromolecular crystallization. Variations that can be introduced in a workshop setting include adjusting systematically the pH, salt and polymer identity, and concentration across the plate.

The focus of this note is on crystallization and on providing a simple and straightforward protocol for gaining a familiarity with handling the sticky and viscous cubic phase. However, having produced crystals, as above, the plates can be used to practice subsequent steps in the *in meso*-based structure determination pipeline. These include harvesting crystals from the viscous mesophase, snap-cooling crystals in liquid nitrogen and using them to practice data collection with a synchrotron-based mini-X-ray beam. Protocols for opening the glass plates and for harvesting and snap-cooling crystals are available online (Li, Boland, Aragao *et al.*, 2012 ▶) and can be used with these *in meso*-grown lysozyme crystals to refine and perfect technique before moving on to valuable target proteins. The behaviour of the test lysozyme crystals and the hosting mesophase should be similar to that experienced when working with genuine target proteins. However, the degree of similarity will depend on the detergent in which the protein is solubilized, assuming it is a membrane protein, and the composition of the precipitant solution. How both impact on mesophase behaviour has been addressed separately (Ai & Caffrey, 2000 ▶; Cherezov *et al.*, 2001 ▶; Misquitta & Caffrey, 2003 ▶; Cherezov *et al.*, 2006 ▶).

While the glass sandwich plates described in this protocol use inexpensive standard microscope slides and cover slips, alternative more X-ray transparent materials can be employed. These include plastics of different types and thicknesses which can be used to screen for crystal diffraction quality directly (*in situ*) on the plate in the lipidic mesophase. Again, lysozyme crystals growing *in meso* are a simple, fast and inexpensive way of performing such tests and of gaining familiarity with what is becoming an increasingly important method for crystal screening and, indeed, for data collection at ambient temperature.

The protocol described was developed for use in setting up *in meso* crystallization trials by hand. It can, of course, be adapted for use with a robot (Cherezov *et al.*, 2004 ▶; Li, Boland, Walsh & Caffrey, 2012 ▶). *In meso* robots are now commercially available and are in use in several laboratories worldwide. Using these robots, crystals can be produced reproducibly within minutes. The protocol described here should prove useful in performing quality control and calibration in such cases.

The fact that lysozyme crystallizes *in meso* is clear evidence that the lipidic mesophase is not just for use with membrane proteins. Thaumatin is another water-soluble protein that readily crystallizes *in meso* (Caffrey, 2000 ▶). There may well be advantages to growing soluble protein crystals *in meso* that relate to the fact that it mimics crystallization in gels and under conditions of microgravity (Caffrey, 2003 ▶). Such conditions stabilize the depletion zone, and minimize the settling of crystals on top of one another and the wafting of contaminants to the growing surface of the crystal, all of which are associated with improved crystal quality. The protocol introduced here can be used to test other water-soluble targets with suitable adjustments made to the protein and precipitant variables in support of crystal growth.

## 4. Conclusions

A quick and easy protocol for crystallizing lysozyme by the *in meso* method, which gives 15–20 µm-sized crystals within an hour, is described. The protocol provides the experimenter with experience in preparing and handling the viscous and sticky cubic mesophase such that the *in meso* method can be used subsequently with confidence, targeting more valuable membrane (and soluble) proteins. The crystals grown *in meso* can, in turn, be used to gain experience with crystal harvesting from the lipidic mesophase and with diffraction data collection using rastering with a micrometre-sized X-ray beam. The protocol should find application in laboratory courses and workshops dealing with macromolecular crystallogenesis, and as a procedure for demonstrating and quantifying the performance of materials, devices, tools and instruments such as crystallization robots and imagers.
